# Dicer- and Bulge Stem Cell-Dependent MicroRNAs During Induced Anagen Hair Follicle Development

**DOI:** 10.3389/fcell.2020.00338

**Published:** 2020-05-14

**Authors:** Neda Vishlaghi, Thomas S. Lisse

**Affiliations:** ^1^Department of Biology, University of Miami, Coral Gables, FL, United States; ^2^Sylvester Comprehensive Cancer Center, Miller School of Medicine, University of Miami, Miami, FL, United States

**Keywords:** microRNA, Dicer, Tarbp2, hair follicle, hair cycle, bulge stem cell

## Abstract

MicroRNAs (miRNAs) are a major class of conserved non-coding RNAs that have a wide range of functions during development and disease. Biogenesis of canonical miRNAs depend on the cytoplasmic processing of pre-miRNAs to mature miRNAs by the Dicer endoribonuclease. Once mature miRNAs are generated, the miRNA-induced silencing complex (miRISC), or miRISC, incorporates one strand of miRNAs as a template for recognizing complementary target messenger RNAs (mRNAs) to dictate post-transcriptional gene expression. Besides regulating miRNA biogenesis, Dicer is also part of miRISC to assist in activation of the complex. Dicer associates with other regulatory miRISC co-factors such as *trans-*activation responsive RNA-binding protein 2 (Tarbp2) to regulate miRNA-based RNA interference. Although the functional role of miRNAs within epidermal keratinocytes has been extensively studied within embryonic mouse skin, its contribution to the normal function of hair follicle bulge stem cells (BSCs) during post-natal hair follicle development is unclear. With this question in mind, we sought to ascertain whether Dicer-Tarpb2 plays a functional role within BSCs during induced anagen development by utilizing conditional knockout mouse models. Our findings suggest that Dicer, but not Tarbp2, functions within BSCs to regulate induced anagen (growth phase) development of post-natal hair follicles. These findings strengthen our understanding of miRNA-dependency within hair follicle cells during induced anagen development.

## Introduction

Mammalian hair follicles undergo cycles throughout life that entail phases of growth (anagen) interspersed by phases of regression (catagen) and then rest (telogen) (Alonso and Fuchs, 2006). The hair cycle requires integration of multiple stimulatory and inhibitory signals in order to properly initiate, maintain or terminate each phase of the cycle (Paus and Cotsarelis, 1999). For example, post-morphogenic hair follicles in the telogen stage receive signals to start the next anagen growth phase to make new hair shafts, and then enter the catagen stage to reset and prepare hair follicle stem cells to enter the ensuing resting stage again (Lisse et al., 2014). As hair follicles mature, cells differentiate to form distinct epithelial layers organized in concentric circles around the centralized hair shaft. There are three major layered compartments of the hair follicle: the innermost layer containing the medulla, cortex and cuticle, the inner root sheath (IRS), and the outer root sheath (ORS) (Paus and Cotsarelis, 1999). The hair cycle and follicle are model systems to investigate intrinsic and extrinsic factors that control stem cell quiescence, activation, and the commitment of stem-cell precursors toward differentiation, and apoptotic death of those cells to repeat the cycle again.

MicroRNAs (miRNAs) are short, highly conserved small non-coding RNA molecules that exist in the genomes of plants and animals (Pong and Gullerova, 2018). miRNAs regulate post-transcriptional mRNA expression typically by guiding effectors to messenger RNAs (mRNAs) at the 3′ untranslated region of the complementary sequences resulting in downregulation of targeted transcripts (Lisse et al., 2013a, b; Papaioannou et al., 2015). Generally, miRNAs are transcribed from DNA sequences to become primary miRNAs, which are then processed into precursor miRNAs (pre-miRNAs) that get further processed to become mature miRNAs (Pong and Gullerova, 2018). The miRNA biogenesis process involves three central factors, namely Drosha, DiGeorge syndrome critical region 8 (DGCR8) and Dicer, while the Argonaut (Ago) proteins interact with mature miRNAs to facilitate post-transcriptional gene silencing (Pong and Gullerova, 2018). In the nucleus, primary miRNAs undergo processing by the Drosha-DGCR8 protein complex to form pre-miRNAs. pre-miRNAs are then exported into the cytoplasm and further processed by Dicer into mature miRNA duplexes. The miRNAs are then loaded onto Ago family proteins to form the miRNA-induced silencing complex (miRISC), whereby target mRNA is degraded or dissociated from the translation machinery.

The functional roles of miRNAs within epidermal keratinocytes have been extensively studied and entail control over cell proliferation, differentiation, migration, development, and epithelial-mesenchymal transitions (Yi et al., 2009; Mardaryev et al., 2010; Yu et al., 2010; Xu et al., 2011). Within embryonic skin of mice, surface ectoderm-specific ablation of core miRNA machinery components *Dicer, Ago1/2, Drosha* or *Dgcr8* disrupts hair follicle development and morphogenesis, and ensuing hair formation (Andl et al., 2006; Yi et al., 2006, 2009; Teta et al., 2012). In particular, constitutive deletion of *Dicer* or *Dgcr8* during embryonic days 14.5–17.5 prevents developing hair follicle enclosure into the underlying dermis to form hair shafts (Andl et al., 2006; Yi et al., 2006, 2009). Additionally, overexpression studies within keratin 14-positive basal epidermal cells have also shown the inhibitory effects of miR-214 on skin and early hair follicle morphogenesis (Ahmed et al., 2014). By and large these findings depict the important role of miRNAs toward embryonic and neonatal development of hair follicles and skin.

Only a few studies have investigated the role of miRNAs during the post-natal period within skin cells. For example, *Dicer* and *Drosha* within keratin 5 (K5)-positive epithelial keratinocytes and/or hair follicle medulla and epithelial stem cells (Joost et al., 2020) of young mice were found to be required for post-natal hair follicle growth and plucking-induced anagen development (Teta et al., 2012). Recently, miR-218-5p was shown to regulate post-natal skin and hair follicle development by induction of the Wnt signaling pathway (Zhao et al., 2019), however, the exact cell types involved remain unclear. Likewise, multiple Dicer-dependent (Lee and Doudna, 2012) miRNAs are expressed in an unique spatial-temporal pattern following the post-natal hair cycle (Mardaryev et al., 2010; Zhao et al., 2019). Nonetheless, whether miRNAs within post-natal hair follicle bulge stem cells (BSCs) – the main source of telogen to anagen transformation – play a functional role during induced anagen development in mice remains unclear. To help bridge this knowledge gap and advance the field, we conditionally ablated *Dicer* and one of its regulatory co-factors, *trans-*activation responsive RNA-binding protein (*Tarbp2*), specifically within BSCs during depilation-induced anagen development. Dicer-Tarbp2 regulates miRNA biogenesis (Pullagura et al., 2018), pre-miRNA cleavage specificity (Lee and Doudna, 2012), and facilitates RNA interference by association with the miRISC loading complex (Lee et al., 2013). Therefore, we hypothesized that global miRNAs and miRISC-mediated RNA silencing are required within BSCs during induced anagen development.

## Results

### Tarbp2 Within Bulge Stem Cells Is Not Required for Depilation-Induced Anagen Development

**FIGURE 1:**
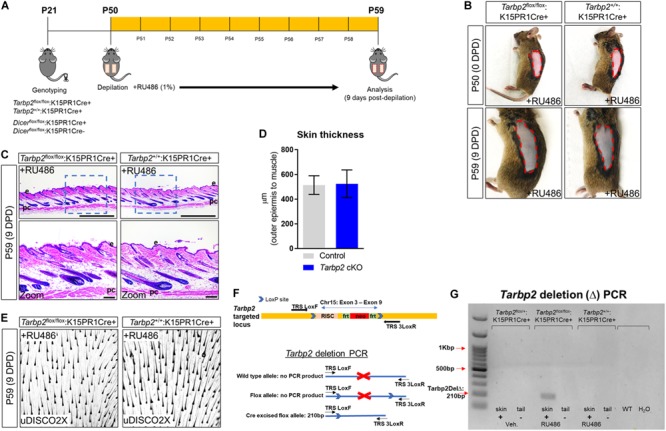
Conditional knock out of *Tarbp2* with hair follicle bulge stem cells. **(A)** Schema outlining the genotyping and conditional knockout of *Tarbp2* and *Dicer* within bulge stem cells. **(B)** Images of the depilated regions of control (*Tarbp2*^+/+^;K15PR1Cre+) and experimental (*Tarbp2*^flox/flox^;K15PR1Cre+) mice at 0 days post depilation (DPD) and 9 DPD. **(C)** Histological analysis of 9 DPD skins in both control (*Tarbp2*^+/+^;K15PR1Cre+) and experimental (*Tarbp2*^flox/flox^;K15PR1Cre+) mice. Boxed region is magnified in the lower panels. Thickness measured from the panniculus carnosus (pc; muscle) to the outer epidermal layer (e). Bars (top panels = 1 mm; bottom panels = 100 um) **(D)** Skin thickness. Distance from the striated muscle layer to the outer epidermal layer (μm). **(E)** Whole mount uDISCO analysis of 9DPD skins in both control (*Tarbp2*^+/+^;K15PR1Cre+) and experimental (*Tarbp2*^flox/flox^;K15PR1Cre+) mice. **(F)** Schema of the targeting construct for generation of *Tarbp2* floxed mice (top panel). Schema of the PCR strategy to determine *Tabp2* ablation efficiency within tissue samples (bottom panel). **(G)** *Tarbp2* deletion PCR results using RU486 pre- and post-treated tissue samples.

To test our hypothesis, we performed hair depilation assays during the resting stage of the hair follicle growth cycle (P50) to promote synchronous initiation of a new hair cycle using inducible *Tarbp2* and *Dicer* floxed mouse lines crossed to keratin 15 (K15) PR1Cre transgenic mice (Figure 1A). This system utilizes the synthetic steroid RU486, the conditional activator of the progesterone receptor Cre recombinase (PR1Cre) fusion protein, to restrict deletion of *loxP* site-flanked *Tarbp2* and *Dicer* sequences strictly within outer BSCs (Joost et al., 2020). By visual inspection, both control *Tarbp2*^+/+^:K15PR1Cre+ and experimental *Tarbp2*^flox/flox^:K15PR1Cre+ mice exhibited hyperpigmentation at 9 days post depilation (DPD) (Figure 1B). Through histological analysis, we observed the expected anagen hair follicles at 9 DPD among control mice (Figure 1C) and observed no histological nor hair follicle growth abnormalities among mutant *Tarbp2*^flox/flox^:K15PR1Cre+ mice (Figure 1D). We also applied an ultimate 3D imaging of solvent-cleared organs (uDISCO) whole-mount clearing method and observed no major difference in the extent of hair growth and hair shaft formation after depilation (Figure 1E). In deletion PCR studies, only RU486-treated skin samples derived from *Tarbp2*^flox/flox^:K15PR1Cre+ mice resulted in *Tarbp2* deletion (Figures 1F,G). Collectively, our results suggest that Tarbp2 regulation of miRISC within BSCs is not essential during induced anagen development of hair follicles.

### Conditional Ablation of *Dicer* Within K15-Postiive Bulge Stem Cells Can Initiate Anagen but Fails to Sustain Proper Development of Hair Follicles

**FIGURE 2:**
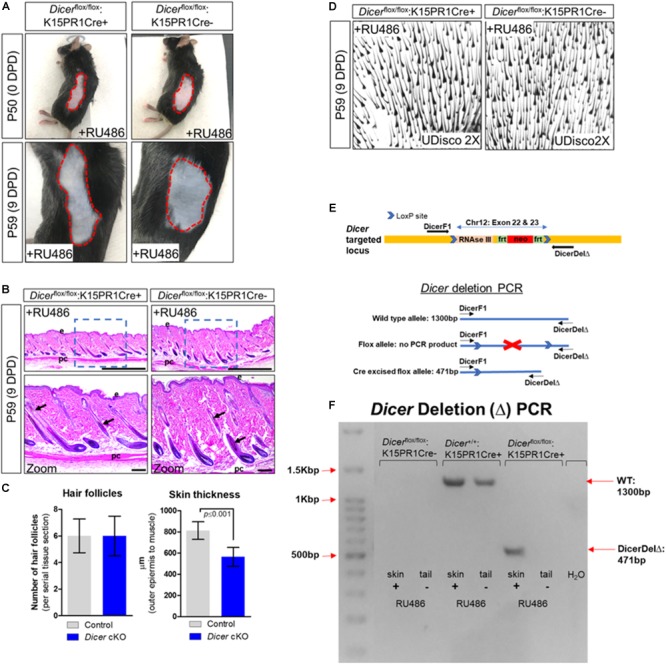
Conditional knockout of *Dicer* within hair follicle bulge stem cells. **(A)** Images of the depilated regions of control (*Dicer*^flox/flox^;K15PR1Cre–) and experimental (*Dicer*^flox/flox^;K15PR1Cre+) mice at 0 days post depilation (DPD) and 9DPD. **(B)** Histological analysis of 9DPD skins in both control (*Dicer*^flox/flox^;K15PR1Cre–) and experimental (*Dicer*^flox/flox^;K15PR1Cre+) mice. Boxed region is magnified in the lower panels. Thickness measured from the panniculus carnosus (pc; muscle) to the outer epidermal layer (e). Bars (top panels = 1 mm; bottom panels = 100 um). **(C)** Hair follicle count and skin thickness analysis. Serial skin sections were assessed from control and Dicer cKO mice (*n* = 12; *p* ≤ 0.001, Student’s *t* test). **(D)** Whole-mount uDISCO analysis of 9DPD skins in both control (*Dicer*^flox/flox^;K15PR1Cre–) and experimental (*Dicer*^flox/flox^;K15PR1Cre+) mice. **(E)** Schema of the targeting construct for generation of *Dicer* floxed mice. **(F)** *Dicer* deletion PCR using RU486 pre- and post-treated tissue samples. Refer to E, schema of the PCR strategy to determine *Dicer* ablation efficiency within tissue sample.

Next, we conditionally ablated *Dicer* within BSCs by generating the *Dicer*^flox/flox^:K15PR1Cre+ mouse model and monitored anagen development after depilation (Figure 1A). By visual observation, both control *Dicer*^flox/flox^:K15PR1Cre− and experimental *Dicer*^flox/flox^:K15PR1Cre+ mice exhibited hyperpigmentation at 9 DPD (Figure 2A) and similar number of anagen hair follicles (Figures 2B–D), suggesting entry into anagen and sufficient hair follicle stem cell lineage specification. However, we observed a small, yet statistically significant decrease in the thickness of skin upon *Dicer* ablation when compared to controls (Figure 2C), suggesting a mild delay in anagen progression (Paus et al., 1999). *Dicer* deletion PCR studies confirmed that only RU486-treated skin samples from *Dicer* mutant mice generated a deletion PCR product (Figures 2E,F).

**FIGURE 3:**
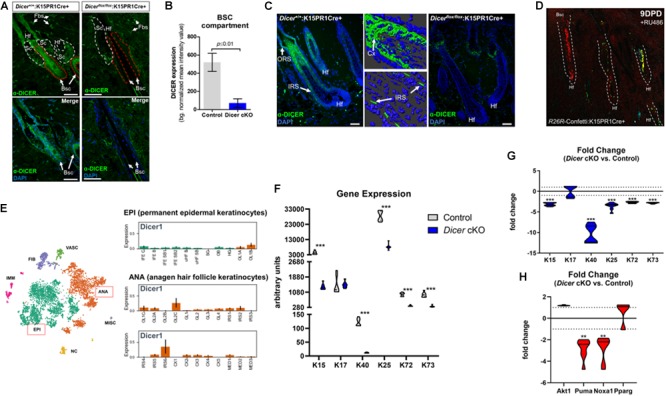
Conditional ablation of Dicer within K15-postiive bulge stem cells impairs hair follicle differentiation. **(A)** Immunofluorescence detection of DICER within various hair follicle compartments of control (*Dicer*^+/+^;K15PR1Cre+) and experimental (*Dicer*^flox/flox^;K15PR1Cre+) skin. Upper panels depict the individual compartments and cell types that express or do not express DICER. Hf (hair follicle), Sc (sebaceous gland compartment; white dash), Fbs (fibroblasts), Bsc (bulge stem cell compartment; red dash). The lower panels highlight only the Bsc and nuclear staining with DAPI. Bars = 50 um (Right panel). **(B)** Quantification of DICER expression from immunofluorescence staining. DICER expression was analyzed within bulge stem cell compartments (*n* = 8; *p* ≤ 0.01, Student’s *t* test) and presented as background normalized mean intensity values. **(C)** Immunofluorescence detection of DICER within the entire hair follicle of control (*Dicer*^+/+^;K15PR1Cre+) and experimental (*Dicer*^flox/flox^;K15PR1Cre+) skin. Right panels depict 3D-rendered models of DICER expression in control hair follicles (Bars = 25 um). ORS (outer root sheath), IRS (inner root sheath), Cx (cortex). Bars = 50 um. **(D)** K15 BSC lineage tracing using the *R26R*-Confetti:K15PR1Cre+ mouse line. **(E)** Dicer mRNA expression at the single cell level during hair growth (anagen) and rest (telogen) in full-thickness skin. Main cell population distribution shown in the upper panel using the T-distributed Stochastic Neighbor Embedding (t-SNE) technique. Permanent epidermis keratinocytes (shown in green, boxed in red) contain subpopulations of resting BSCs and epidermal keratinocytes. The anagen hair follicle keratinocytes (shown in orange, boxed in red) contain subpopulations of cells undergoing growth and differentiation. EPI (permanent epidermal keratinocytes), ANA (anagen hair follicle keratinocytes), VASC (vascular cells), FIB (fibroblast-like cells), IMM (immune cells), NC (neural crest-derived cells), MISC (miscellaneous). The lower panel shows Dicer1 expression in log2 scale on the *y*-axis within subpopulation of EPI and ANA cells. EPI cells consist of interfollicular epidermis (IFE) basal cycling (C), basal (B) and suprabasal (SB) cells, upper hair follicle (uHF) basal (B) and suprabasal (SB) cells, sebaceous glands (SG), outer bulge (OB), and hair germ (HG) cells. ANA cells consist of outer layer (OL) cells, germinative layer (GL) cells, inner root sheath (IRS) cells, cortex/cuticle (CX) cells, and medulla (MED) cells. Data derived from: (Joost et al., 2020). **(F)** Arbitrary keratin gene expression using real-time PCR. Gene expression presented as arbitrary units for individual genes and samples (*n* = 3; ***p ≤ 0.001, two-way ANOVA). **(G)** Fold change in keratin gene expression. Fold change in gene expression comparing *Dicer* cKO versus control. Dotted line is no change in gene expression (*n* = 3; ***p ≤ 0.001, two-way ANOVA). **(H)** Fold change in apoptosis, pro-survival and sebaceous gland gene expression. Fold change in gene expression comparing *Dicer* cKO versus control. Dotted line is no change in gene expression (*n* = 3; **p ≤ 0.01, two-way ANOVA).

To further validate *Dicer* ablation within BSCs, we performed immunofluorescence staining for DICER using an antibody that specifically recognizes exons 22–23 of DICER (i.e., the region of *Dicer* which was excised). We observed a statistically significant (*p* ≤ 0.001, Student *t* test) decrease in background-normalized DICER immunoreactivity within the BSC compartments of mutant mice when compared to control (Figures 3A,B). Importantly, we also observed decreased DICER expression within BSC progeny of individual mutant hair follicles, as well as a decrease in overall cellularity within mutant hair follicles (Figure 3C). The cellular decrease in DICER expression throughout mutant hair follicles was likely due to hair follicle resolve to monoclonality as also shown by the K15PR1Cre+:R26R-Confetti reporter line (Figure 3D). As Dicer is the major enzyme involved in pre-to-mature miRNA processing (Pong and Gullerova, 2018), the production of mature miRNAs within hair follicles is likely compromised in our conditional knockout animals. In control hair follicles, we observed DICER-positive cells in the upper ORS, combined with increased presence within the IRS, cortex (Cx) and preCX. Furthermore, we compared our DICER expression pattern within the most recent and comprehensive temporal and spatial single cell transcriptomic analysis of hair follicle cells in either the resting or anagen growth stage (Joost et al., 2020). The DICER immunostaining pattern in the control animals was consistent with the single cell-RNAseq data showing Dicer mRNA expression specifically within a subset of Cx/IRS as well as ORS cells within anagen hair follicles (Figure 3E), suggesting the cell types of miRNA dependency (Joost et al., 2020).

### Conditional Ablation of *Dicer* Within K15-Postiive Bulge Stem Cells Disrupts Keratin Gene Expression in the Bulge, IRS and Cortex Cell Reserves, but Not ORS Populations

Next, we used quantitative real-time PCR to ascertain potential changes in gene expression within specific subsets of hair follicle cells upon BSC-specific *Dicer*-deletion to account for the observed skin phenotype. Keratins are intermediate filament-forming proteins and represent major cellular constituents within hair follicular cells. Keratins are expressed in a differentiation-dependent manner, thus are useful markers to study epithelial dynamics (Fuchs, 1995). Here we appraised differential keratin gene signatures expressed within murine BSCs (K15), ORS (K17), IRS (K25/72/73), and cortex/cuticle (K40) cells (Joost et al., 2020). We observed a statistically significant decrease in K40 and K15 mRNA levels among *Dicer*^flox/flox^:K15PR1Cre+ experimental mice compared to controls (Figure 3F). This translated into a dramatic 10 and 3-fold-decrease in transcript levels of K40 and K15, respectively, after *Dicer* ablation (Figure 3G). As K40 is one of the last keratins to be expressed by cortex/cuticle cells during the differentiation of hair follicles (Langbein et al., 2007), these results suggests that their differentiation is mediated by *Dicer*. Furthermore, a decrease in K15 suggests that the long-term maintenance of BSC reserves post-anagen initiation are mediated by *Dicer* as well.

Interestingly, K17 transcript levels were unchanged between *Dicer*^flox/flox^:K15PR1Cre+ experimental and control mice (Figures 3F,G), suggesting selective actions of Dicer within hair follicular cells. In mice, K17 is expressed spatially within ORS compartments at all stages of the hair cycle (e.g., within the early suprainfundibular and isthmus, as well as the later-stage proximal region of the ORS) (Panteleyev et al., 1997; Joost et al., 2020). These results suggest that although Dicer is expressed in ORS cells, Dicer-mediated miRNAs play a limited functional role within K17-postiive ORS cells during induced anagen development. In addition, this may also reflect a novel “pro survival” role of K17 within *Dicer*-ablated ORS cells (Oshima, 2002; Tong and Coulombe, 2006). In addition, we appraised gene signatures of IRS cells and observed statistically significant decreases in K25/72/73 transcript levels within *Dicer*^flox/flox^:K15PR1Cre+ experimental skin samples compared to controls (Figures 3F,G). As Dicer is also expressed within IRS cells (Figure 3C; Joost et al., 2020), it appears to play a functional role within subsets of these cells during induced anagen development. Based on the overall level differences in gene expression, our results suggest that Dicer plays a larger role within K40-postiive keratinocytes during induced anagen development.

### Conditional Ablation of *Dicer* Within K15-Postiive Bulge Stem Cells Does Not Enhance Apoptosis or Sebocyte Formation During Induced Anagen Development

Previous studies that utilized a K5-mediated *Dicer*-deletion mouse model reported an enhancement of apoptosis as a mode for hair degeneration during induced anagen development (Teta et al., 2012). To test if apoptosis was affected in our system, we appraised several markers of apoptosis and cellular survival pathways using our *Dicer*^flox/flox^:K15PR1Cre+ experimental and control skin samples. We had previously utilized both ATF4-mediated p53 upregulated modulator of apoptosis (Puma) and NADPH oxidase activator 1 (Noxa1) as valid markers to confirm and study the apoptosis-driven catagen stage of the post-morphogenic hair cycle (unpublished data). Furthermore, Puma is a *bona fide* apoptosis marker expressed in hair follicles (Vesela and Matalova, 2015). We observed no significant difference in the transcript levels of pro-survival Akt1 (also called protein kinase B) between *Dicer* mutant and control skin samples after induced anagen development (Figure 3H). On the other hand, we observed a statistically significant ∼−2.8- and ∼−2.7-fold decrease in pro-apoptotic Puma and Noxa1 mRNA levels, respectively, among *Dicer* mutant skin samples compared to controls (Figure 3H). These results signify a possible delay in the hair cycle after *Dicer* deletion compared to control hair follicles that are advancing toward regression. Lastly, select miRNAs have been shown to regulate the “stemness” of hair follicle stem cells toward hair, epidermal and sebaceous gland differentiation (Zhang et al., 2011).

To better understand whether Dicer-dependent miRNAs play a role in dictating plasticity among BSCs toward different lineages of skin (Snippert et al., 2010; Zhang et al., 2011), we appraised the transcript level of peroxisome proliferator-activated receptor gamma (Ppar-γ or Pparg), an important mediator of lipid metabolism in sebaceous gland cells. In our comparison between *Dicer*^flox/flox^:K15PR1Cre+ experimental and control skin samples, we observed no significant difference in Pparg mRNA levels (Figure 3H), indicating that *Dicer* ablation within BSCs does not affect the pool of sebocytes after induced anagen development. Overall, in the absence of functional *Dicer*, these data provide a mode for delayed induced anagen development that does not involve induction of apoptosis nor alterations in the “stemness” of BSCs.

## Discussion

MiRNAs were first discovered in worms and found to control biological processes through a diverse set of mechanisms including gene, transcriptional, post-transcriptional and translational regulation (Lee et al., 1993; Lisse et al., 2013a, b). miRNAs and their targets form diverse regulatory networks involved in different cell lineages of hair follicles to support their initial development and post-natal growth and maintenance (Zhang et al., 2011). A simple explanation why Dicer appeared to have a more pronounced functional impact after the onset of anagen development might be due to its increased temporal and spatial expression within hair follicle cells over time. This was evident in the single cell sequencing data generated by Joost et al. (2020), whereby Dicer transcripts elevated within subpopulations of follicular keratinocytes transitioning from the telogen to the anagen phase (Figure 3E). Moreover, the regulation of Dicer is not known during these transitions across the cell types and hair follicle stages. This might be reminiscent of the lineage-specific transcriptional regulation of *Dicer* within melanocytes by MITF, a basic helix-loop-helix leucine zipper transcription factor, identified through analysis of differential miRNA expression profiles over time (Levy et al., 2010).

Previous studies have underscored the role of miRNAs during skin development and post-natal skin maintenance by manipulating specific cell types. Global depletion of miRNAs will result in distinct skin and hair phenotypes depending on in which cells they are ablated. For example, Teta et al. (2012) ablated the essential miRNA biogenesis enzymes Drosha and Dicer within K5-positive cells in mouse skin. In those studies, a severe hair loss phenotype was observed due to, in part, increased apoptosis in the rapidly proliferating matrix cell population of hair follicles. The authors also reported that there were no differences in the BSC pool between the mutant and control animals, suggesting a K5-specific later-stage phenotype. K5 is expressed by cells within multiple compartments of the skin and hair follicle, including BSCs, inner medulla cells and interfollicular epithelial keratinocytes of the basal epidermal layer of skin (Joost et al., 2020). Dicer is also expressed in late-developing medulla cells (Figure 3E), however, it is unclear if there is any link between the medulla cells and the phenotype observed after K5-specific *Dicer* ablation. Teta et al. (2012) concluded that rapidly proliferating matrix keratinocytes depend on global miRNAs, otherwise they experience enhanced apoptosis. However, given the expression pattern of K5, it is possible that the phenotype may be due to miRNA-dependent interfollicular epidermal defects as observed upon K14-driven epidermal-specific *Dicer* ablation (Andl et al., 2006). On this note, the K14-specific *Dicer* mutants also exhibit severe hair follicle phenotypes, further highlighting the specific functions *Dicer* plays within skin compartments, and that epidermal defects can effect hair follicle development.

Teta et al. (2012) also investigated the role of *Dicer* specifically within K15-postive BSCs during induced anagen development. The authors utilized a lineage tracing approach to decipher if loss of *Dicer* within K15-positive BSCs affected proper anagen development, whereby no defects were reported. The authors concluded that *Dicer* within K15-positive cells was dispensable for induced anagen development since *Dicer*-deleted BSCs were able to contribute cells to select hair follicle compartments over time (Teta et al., 2012). However, there are several limitations to those studies. First, the lineage tracing studies were not quantified, therefore it remains inconclusive about the extent of K15-derived *Dicer*-less progeny recruited to hair follicle compartments. Second, the complete hair follicle profiles in the tissue sections were lacking, thus the results do not provide the actual potential of BSC-precursors upon *Dicer* deletion. And third, and possibly most important, the extent of follicular defect was not adequately assessed as the potential hair follicle stage and molecular phenotypes were not investigated. Therefore, based on the Teta et al. studies alone, it remains inconclusive whether *Dicer* ablation within BSCs affects induced anagen hair follicle development.

In this study, we provide more clarity to the role of *Dicer* and *Tarbp2* within K15-postiive BSCs during induced anagen development. Within the limit of our studies (Pullagura et al., 2018), we found that *Tarbp2* does not play a functional role in the regulation of induced anagen development. It is possible that co-regulatory redundancy of Tarbp2 by other Dicer-associated co-factors may help define miRNA specificity within BSCs instead (Pullagura et al., 2018). When *Dicer* was ablated strictly within K15-postive BSCs compared to K5-postive cells (Teta et al., 2012), we observed milder hair, hair cycle and skin phenotypes after induced anagen development. The K5-dependent *Dicer* deletion studies reported an enhancement in apoptosis as a mode for severe hair degeneration (Teta et al., 2012). Upon K15-mediated *Dicer* deletion, we uncovered a hair cycle stage delay depicted by morphological changes in the skin, and downregulation of key gene signatures for follicular differentiation and apoptosis (Figure 3). Interestingly, our observations are consistent with the role of miRNAs in other stem cell models, including that of male testicular spermatogonia stem cells (SSCs). In testes, *Dicer* ablation causes a failure in haploid differentiation of SSCs resulting in abnormal spermatozoa and infertility (Korhonen et al., 2011).

In addition, we report a decrease in gene expression markers for BSCs and their progeny cells, such as IRS cells within *Dicer* mutant animals after induced anagen development (Figures 3F,G). These findings suggest the importance of Dicer for these cells; however, we do not know yet the identities of those responsible miRNAs and targets. miR-24 is highly expressed in the IRS and plays a functional role in hair follicle differentiation (Amelio et al., 2013). To date, miR-24 is the only miRNA found to be expressed in the IRS and directly represses the hair keratinocyte stemness regulator *Tcf-3* (Amelio et al., 2013). In the same study, miR-24 transgene overexpression led to abnormal hair follicle development by way of premature differentiation. Therefore, the potential lack of miR-24 within our BSC-specific *Dicer* deletion studies may contribute to the delay in the hair cycle and keratin expression within hair follicle keratinocyte sub populations.

Zhang et al. (2011) showed that miR-125b is expressed in various hair follicle stem cell compartments, and is downregulated in epidermal and stem cell progeny after onset of anagen. Through overexpression studies, the authors suggested that miR-125b was indispensable for anagen onset by maintaining hair follicle stem cell “stemness” by acting as a repressor of skin cell differentiation. However, from these studies it was unclear in which stem cell populations (e.g., K15+, Lgr6+, Lgr1+) miR-125b might play a functional role. In addition, the authors identified putative miR-125b targets that could explain the switch in “stemness” to epidermal, sebaceous gland and hair differentiation (Zhang et al., 2011). However, it is possible that miRNAs are not crucial for the onset of anagen from the BSC perspective. First, we show formation of the correct number of hair follicles and proper gene expression for sebocytes upon conditional *Dicer* deletion within BSCs. And second, Teta et al. (2012) has also shown that the onset of anagen was not impaired when *Dicer* was ablated in K15-positive BSCs. Given the proposed importance of miR-125b in maintaining the “stemness” of hair follicle stem cells (Zhang et al., 2011), one would expect that in the absence of *Dicer* maturation of hair follicles is accelerated. However, our results suggest a delay in the hair cycle upon *Dicer* ablation in BSCs. To reconcile this difference, it is possible that miR-125b functions within non-BSC stem cell compartments such as the isthmus, which is positioned atop the BSC compartment in order to dictate “stemness.” In line with this hypothesis, Lgr6-postive hair follicle stem cells that constitute the isthmus are known to give rise to sebaceous glands, for example (Snippert et al., 2010). In our studies we show that sebaceous glands are not affected by *Dicer* deletion within BSCs, and once again may reflect in which particular cell types miRNAs dictate function.

Lastly, in terms of disease, although most of the allelic variants of *Dicer* are associated with several tumor types, it is currently unclear if variants exist that play a role in BSC-mediated hair follicle maturation-related diseases including alopecia (OMIM 606241). It is also unclear, given the mild hair phenotype associated with *Dicer* ablation within BSCs, whether other biological responses such as those involving skin injury or chronic wounding, are affected. Given the reduction in the BSC pool after BSC-specific *Dicer* deletion, and the contribution of BSC progeny to damaged epidermis after injury (Ito et al., 2005), it is possible that Dicer may play a role in the wound repair process in mice as well.

## Materials and Methods

### Mouse Models and Breeding

*Dicer*-null conditional (*Dicer*1*^TM 1Bdh^*/J) mice (referred to as *Dicer*^floxed^; JAX stock no. 0063661) and *Tarbp2*- Null conditional (*Tarbp2^TM 1.1Dzw^*) mice (referred to as *Tarbp2*^Floxed;^MGI: 5645258) were received from the Jackson laboratory. The Tg(Krt1-15-cre/PGR)22Cot line (referred to as *K15*-PR1Cre; JAX stock no. 005249) was on a mixed C57BL/6 and SJL background also obtained from Jackson Laboratory and was used for generation of RU486 (Sigma; M8046)-induced, Cre recombinase-mediated gene deletions within BSCs of hair follicles. *Dicer*^flox/flox^ (*Dicer*^fl/fl^) and *Tarbp2*^flox/flox^ (*Tarbp2*^fl/fl^) mice were bred with K15-Cre line to achieve cell-specific knockout of targeted regions within BSCs of hair follicles. Lineage tracing was performed using the *Gt(ROSA)26Sor^TM 1(CAG–Brainbow2.1)Cle/J^* (also called the R26R-Confetti line) mice obtained from the Jackson Laboratory (R26R-Confetti; JAX stock no. 013731). For linage tracing, we generated the K15PR1Cre+:R26R-Confetti reporter line and induced recombination with RU486 topical treatment at P50-P53 of age. The following day, we depilated the fur of the animals and waited 9 days (P63) to harvest the skin for analysis of BSC progeny using the confocal microscope. All animals were maintained in a 12-h light (6am to 6pm) and 12-h dark cycle vivarium in the Research Animal Facility at the University of Miami. Animals were provided acidified tap water through filter bottle and irradiated pelleted 2018 Teklad global 18% protein rodent diet from ENVIGO. In timed pregnancy crosses, pups were dated based on the presence of vaginal plugs and by noting the delivery of newly born pups. All animal studies were approved by the University of Miami Institutional Animal Care and Use Committee (IACUC) protocols.

### Genotyping and Recombination Detection

All genotyping proceeded by using tail tip excision/partial amputation under the age of 21 days. Dicer floxed allele was genotyped by using primers DicerF1 (CCTGACAGTGACGGTCCAAAG) and DicerR1 (CATGACTCTTCAACTCAAACT). PCR product for genotyping PCR was 420-bp band for Dicer and a 351-bp band for wild type allele. The deletion was genotyped by using primers DicerF1 and DicerDel (CCTGAGCAAGGCAAGTCATTC). PCR product for deletion PCR was a 471-bp band for deletion and a 1300-bp band for the wild type allele. To assay floxed versus wild type allele in Tarbp2 animals, we used TRS-loxF (CAGAAGCACAGCAGGAACAA) and TRS-loxR (CGTGATATGCACAGCCCACT) primers. PCR product for genotyping PCR was 180-bp band for the floxed and a 130-bp band for the wild type allele. Deletion allele was detected by using primers TRS-loxF and TRS-3loxR (CAAAACCACTTCCCCATGTT). All mice genotyping was based on established protocols listed on the JAX website for each stock animal. PCR program was run according to recommendations: 95°C for 5 min, and for 35 cycles 95°C for 15 s, 60°C for 15 s, and 72°C for 10 s for Dicer genotyping and deletion PCR; 94°C for 3 min, for 35 cycles 94°C for 30 s, 61.7°C (59°C annealing for delete allele) for 30 s and 72°C for 20 s for Tarbp2 PCR.

### Conditional Knockout Studies and Hair Depilation Assay

Under general anesthesia (inhalation isoflurane), 50-day-old animals were subjected to hair depilation of two separate left and right side of the dorsal area by using Wax Strips after hardening. All procedures were performed using sterile instruments and aseptic conditions. Mice received topical daily treatments of 1% RU486 (Sigma; M8046) in acetone, or acetone only (vehicle), over the depilated area for 9 days. Nine days after depilation, animals were sacrificed, and skin samples were collected for analysis.

### Ultimate DISCO (uDISCO) Whole Mount Passive Clearing Technique for Skin

To visualize thick skin tissue and hair morphology, we applied the organic solvent-based uDISCO clearing method. Greater than 3 mm-by-3 mm skin tissues were fixed in 4% paraformaldehyde at 4°C overnight. Samples were washed with 0.1 M phosphate buffered saline and subjected to a tert-butanol series (70–100%; Sigma-Aldrich, 36053) for gradient dehydration for 2–12 h. Next, dichloromethane (Sigma-Aldrich, 270997) was replaced as a pure solution for the delipidation step for 1 h at room temperature. A refractive index matching solution was prepared by mixing BABB (benzyl alcohol + benzyl benzoate 1:2; Sigma-Aldrich, 24122 and W213802) and DPE (diphenyl ether) (Sigma-Aldrich, 240834) at a BABB:DPE ratio of 10:1 (vol/vol). The samples were reacted with the BABB:DPE mixture until they became optically transparent.

### Histological, Histomorphometric and Immunofluorescence Analysis

Skin tissues (1 × 1 cm^2^) from the lower trunk region were dissected from mice and incubated 2–4 h in 4% paraformaldehyde in phosphate buffered saline (pH 7.4) at 4°C and transferred to 70% ethanol, before embedding in paraffin wax. Sections at 7 μm were stained with hematoxylin and eosin (H&E) by The University of Miami Histology Core Services. For immunofluorescence studies, skin tissues (1 × 1 cm^2^) were dissected from mice and incubated 2–4 h in 4% paraformaldehyde in phosphate buffered saline (pH 7.4) at 4°C and subjected to graded sucrose treatments (15–30%) for cryoprotection. These tissues were embedded face down along the midline in optical cutting temperature embedding medium (Histolab; 45830). Cryosections were made using a Leica CM 1850 Cryostat. Sections at 10 μm were incubated with 0.4% Triton X-100 in phosphate buffered saline for 30 min at room temperature to reduce background staining. Tissue sections were directly blocked in phosphate buffered saline containing 5% normal horse serum for 30 min at room temperature. Then incubated with endogenous mouse immunoglobulin G blocking solution 1:10 in phosphate buffered saline (Unconjugated AffiniPure Fab Fragment Goat Anti-mouse immunoglobulin G (H + L); Jackson ImmunoResearch Labs, 115-007-003) for 1 h at room temperature. Sections were incubated with primary antibodies at 1:200 dilution for 30 min at room temperature; antibodies used in this study included Dicer (BioLegend. 820201). Following 3x washes for 2 min each in phosphate buffered saline-Tween-20, the sections were incubated at room temperature for 20 min with corresponding species-specific 1:2000 secondary antibodies (Alexa series, Life Technologies). Following 3x washes in phosphate buffered saline-Tween-20, sections were mounted with Vectashield medium containing 4′,6-diamidino-2-phenylindole (DAPI; Vector Laboratories; H-1200-10) for nuclei staining. Negative controls were included that had either no primary or secondary antibodies in the blocking buffer. Immunofluorescence microscopy was performed using a Zeiss Observer 7 ApoTome2 unit. Images were captured from BSCs compartment for DICER expression and normalized to acellular dermal regions. Means of expression intensity of individual follicles were compared between control and mutant animals (*n* = 8). Imaris (Bitplane) was used to generate 3D rendered models of Dicer expression. For hair follicle counts (i.e., hair follicles per 2.5 mm tissue; *n* = 12 sections) and skin thickness measurements (i.e., distance from panniculus carnosus (muscle) to the outer epidermis; *n* = 12 sections), serial skin sections were analyzed between control and cKO mice. Two-tailed unpaired *t* tests were performed between control and cKO data sets using Prism (GraphPad) where the *p* value summaries were depicted as ^∗∗∗^*p* ≤ 0.001, ^∗∗^*p* ≤ 0.01, and ^∗^*p* ≤ 0.05.

### Quantitative Real-Time RT-PCR (qPCR) Analysis

RNA was prepared using the PureLink RNA Mini kit (ThermoFisher Scientific). cDNA was synthesized using 200 ng total RNA with the ProtoScript^®^ First Strand cDNA Synthesis kit (New England Biolabs; M0368) utilizing random hexamers. All cDNAs were amplified under the following conditions: 95°C for 10 min to activate AmpliTaq Gold^®^ Polymerase; followed by 40 cycles of 95°C for 15 s and 60°C for 1 min with an internal ROX reference dye. qPCR analysis was performed on a QuantStudio 3 Real-Time instrument (ThermoFisher Scientific) utilizing the Power SYBR^TM^ Green PCR Master mix (ThermoFisher Scientific; 4367659). Target genes were normalized to beta actin mRNA expression. For the primer design, the mouse genome sequence coverage assembly GRCm38.p6 was utilized from the Genome Reference Consortium. K17 primers: Forward 5′GGAGCAGCAGAACCAGGAAT3′ and reverse 5′ TCGCGGGAGGAGATGACC3′. K15 primers: Forward 5′AGGAGGTGGCGTCTAACACAGA3′, and reverse 5′CATGCTGAGCTGAGACTGCAAC3′. K40 primers: Forward 5′TGCCAGACTGAGATGTTGGA3′, and reverse 5′GCCCCTGTACGTGTTGATCT3′.

Beta actin primers: Forward 5′CCAGTTCGCCAT GGATGACGATAT3′, and reverse 5′GTCAGGATACCTCT CTTGCTCTG3′.

K25 primers: Forward 5′GGCCAGAAGCTGGAATATGA3′, and reverse 5′CCACTATGGCTTTGACTGGA3′.

K72 primers: Forward 5′GAGATCGCCACCTACAGGAA3′, and reverse 5′CCACAGCTACCCTTGGTCTT3′.

K73 primers: Forward 5′GAGATCGCCACCTACAGGAA3′, and reverse 5′GCTACCCTTGGTCTTCACCTC3′.

PPARG primers: 5′TCACAATGCCATCAGGTTTG3′, and reverse 5′TCCGTTGTCTTTCCTGTCAA3′.

Noxa1 primers: 5′CCCAGGCGATACCTAAAACA3′, and reverse 5′TGGATGCCAGCAAACTATCA3′.

Puma primers: 5′GCCCAGCAGCACTTAGAGTC3′, and reverse 5′TGTCGATGCTGCTCTTCTTG3′.

Akt1 primers: 5′CCCTTCTACAACCAGGACCA3′, and reverse 5′TGGGCTCAGCTTCTTCTCAT3′. Data are presented as fold induction of *Dicer* cKO compared to control samples normalized to beta actin mRNA levels (i.e., the comparative CT Livak method (Livak and Schmittgen, 2001). Data also presented as arbitrary values derived directly from the dCT values (2^–dCT^ × 10^4^). Melting curve analysis was performed for all primers to eliminate those that yielded primer-dimers. As the delta Ct (dCT) values are measures that are proportional to log expression, a *t*-test using two groups (control and mutant; *n* = 6; in triplication) of dCT values was used to generate the *p*-values.

## Data Availability Statement

The data supporting the results reported in this article will be provided upon reasonable request.

## Ethics Statement

The animal study was reviewed and approved by the Institutional Animal Care and Use Committee University of Miami.

## Author Contributions

NV conducted the experiments and edited the manuscript. TL was involved in the conceptualization, project administration, formal investigation, methodology and experimentation, analysis of the data, and writing of the manuscript.

## Conflict of Interest

The authors declare that the research was conducted in the absence of any commercial or financial relationships that could be construed as a potential conflict of interest.
